# Glycosylated Hemoglobin in Relationship to Cardiovascular Outcomes and Death in Patients with Type 2 Diabetes: A Systematic Review and Meta-Analysis

**DOI:** 10.1371/journal.pone.0042551

**Published:** 2012-08-09

**Authors:** Yurong Zhang, Gang Hu, Zuyi Yuan, Liwei Chen

**Affiliations:** 1 First Affiliated Hospital of Medical School, Xi’an Jiaotong University, Xi’an, Shaanxi, China; 2 Chronic Disease Epidemiology Laboratory, Pennington Biomedical Research Center, Baton Rouge, Louisiana, United States of America; 3 Department of Epidemiology, Merck Sharp and Dohme Corp, Whitehouse Station, New Jersey, United States of America; College of Pharmacy, University of Florida, United States of America

## Abstract

**Background:**

Chronic hyperglycemia in type 2 diabetes increases the risk of microvascular events. However, there is continuing uncertainty about its effect on macrovascular outcomes and death. We conducted a meta-analysis of prospective studies to estimate the association of glycosylated hemoglobin level with the risk of all-cause mortality and cardiovascular outcomes among patients with type 2 diabetes.

**Methodology/Principal Findings:**

We systematically searched the MEDLINE database through April 2011 by using Medical Subject Heading search terms and a standardized protocol. We included prospective cohort studies that reported data of glycosylated hemoglobin level on the risk of incident cardiovascular events and all-cause mortality. Relative risk estimates (continuous and categorical variables) were derived or abstracted from each cohort study. Twenty six studies were included in this analysis with a mean follow-up rang of 2.2–16 years. The pooled relative risk associated with a 1% increase in glycosylated hemoglobin level among patients with type 2 diabetes was 1.15 (95% CI, 1.11 to 1.20) for all-cause mortality, 1.17 (95% CI, 1.12 to 1.23) for cardiovascular disease, 1.15 (95% CI, 1.10 to 1.20) for coronary heart disease, 1.11 (95% CI, 1.05 to 1.18) for heart failure, 1.11 (95% CI, 1.06 to 1.17) for stroke, and 1.29 (95% CI, 1.18 to 1.40) for peripheral arterial disease, respectively. In addition, a positive dose-response trend existed between glycosylated hemoglobin level and cardiovascular outcomes.

**Conclusions/Significance:**

Chronic hyperglycemia is associated with an increased risk for cardiovascular outcomes and all-cause mortality among patients with type 2 diabetes, likely independently from other conventional risk factors.

## Introduction

Type 2 diabetes continues to be one of the most common and important public health crises worldwide. It has been estimated that the global health expenditure on diabetes is at least $376 billion in 2010 and will be $490 billion in 2030 [1]. Type 2 diabetes is a major risk factor for cardiovascular disease (CVD). Patients with type 2 diabetes have a 2–4 times higher risk of CVD mortality than those without diabetes [2], [3]. CVD accounts for approximately 70% of death among patients with type 2 diabetes [4], [5].

The key risk factor associated with diabetes complications is poor glycemic control [6]. Growing evidence has linked chronic hyperglycemia to microvascular complications [7]–[9]. Although improving glycemic control has been demonstrated to reduce microvascular complications in patients with type 2 diabetes [9], [10], the relationship between glycosylated hemoglobin (GHb) level and macrovascular complications and all-cause mortality is still uncertain. In three meta-analyses [11]–[13] of published randomized controlled trials (RCTs), intensive glycemic control showed positive effects on some cardiovascular outcomes, but did not reduce the risk of death from CVD and all causes. However, these studies were constrained by inherent limitations of the clinical trials, which might have been underpowered to show clinical benefits - especially if event rates were lower than expected or the studies had a high lost follow-up rate [14].

A meta-analysis of 10 prospective studies evaluating the association of GHb level with CVD risk among patients with type 2 diabetes has found that every 1% increase in GHb was associated with an 18% increase in the risk of CVD events [15]. However, this meta-analysis did not estimate the association of GHb level with the risk of all-cause mortality. Moreover, among all prospective studies included, only two studies had a baseline sample size greater than 2,000 [16], [17]. In recent years, several high quality and large prospective studies have assessed the association of GHb level with the risks of CVD outcomes and/or all-cause mortality in patients with type 2 diabetes, but the results are inconsistent. With respect to all-cause mortality, a U-shape association [18] and a non-linear positive association [19] have been reported in these cohort studies. For CVD outcomes, most studies reported a positive association [19]–[24], whereas one study found no relation [25]. In addition, some data from previous cohort studies have been updated recently [17], [26], [27].

To clarify whether lowering long-term GHb level can reduce the risks for CVD outcomes and all-cause mortality, we performed a systematic review and meta-analysis with the most updated prospective data to evaluate the association of GHb level with the risks of incident CVD outcomes and all-cause mortality in patients with type 2 diabetes.

## Methods

### Data Sources and Searches

We searched the MEDLINE database for articles published in English from January 1974 to April 2011 by using Medical Subject Heading (MeSH) terms *cardiovascular diseases; coronary heart disease; heart failure; stroke; peripheral arterial disease; all-cause mortality*; and *diabetes mellitus, type 2*, as well as *glycemic control*, and *glycosylated hemoglobin* or *HbA1*c. We also performed a manual searching of references cited by original studies and relevant review articles and queried experts to identify any additional studies. This search provided 3123 articles, which were further screened for inclusion from abstracts or full texts.

**Figure 1:**
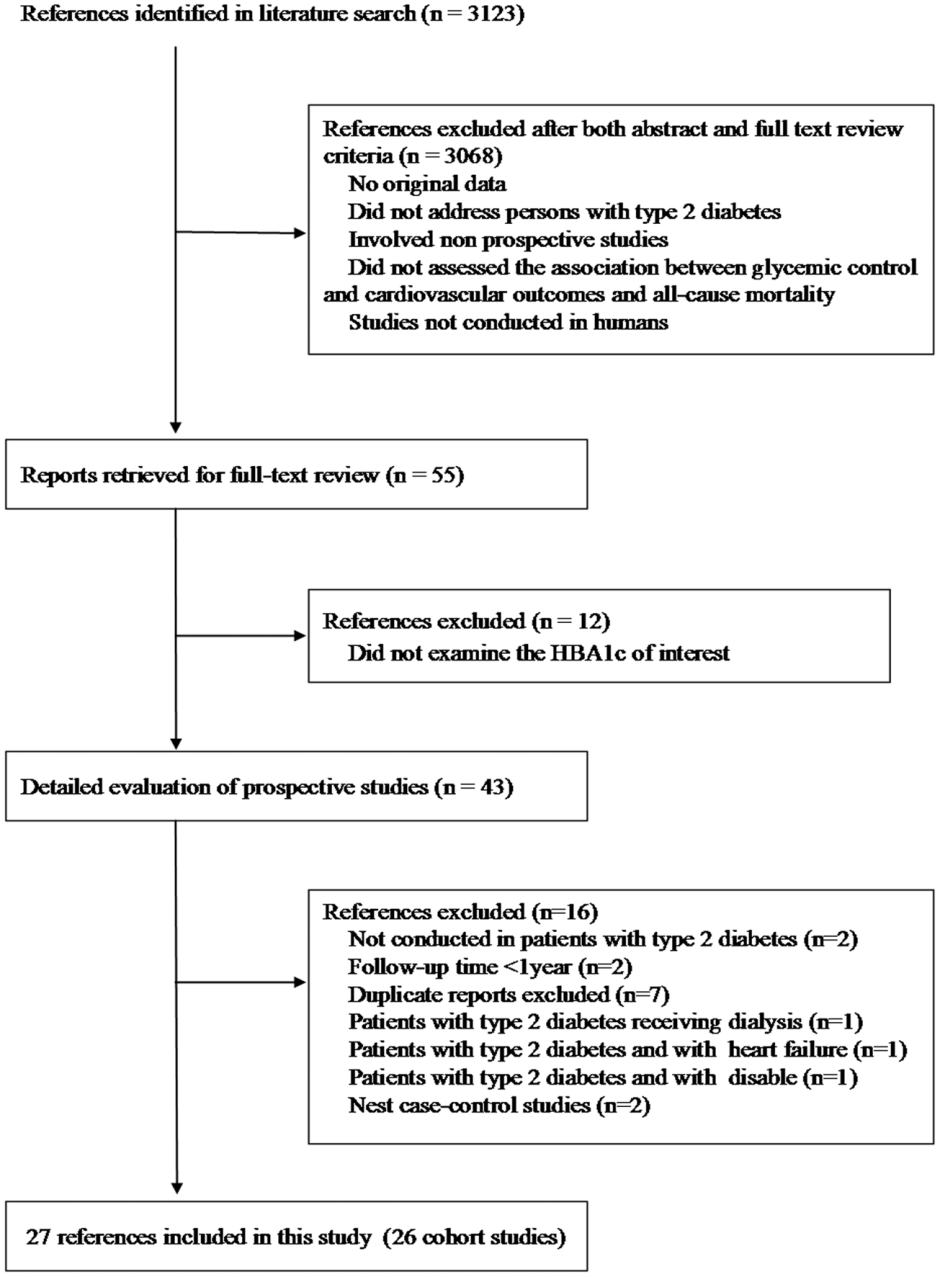
Flow diagram of studies assessed and included.

### Study Selection

We selected the studies based on the following conditions: 1) study design: prospective cohort studies; 2) study population: patients with type 2 diabetes; 3) studies reported at least one of the outcomes of interest: cardiovascular outcomes (CVD, CVD mortality, coronary heart disease (CHD), fatal CHD, heart failure, and stroke), peripheral arterial disease (PAD), and all-cause mortality; and 4) studies reported a measure of GHb (HbA1_C_, HbA_1_, and total GHb). We first identified 55 full-text articles and then excluded some if they 1) had no original data (review, editorials, meta-analyses), 2) involved non prospective analysis (e.g., nest case–control studies), 3) had follow-up time <1 year, 4) included patients with type 2 diabetes receiving dialysis, or with heart failure, or with disability, or 5) were duplicate publications. If separate articles from the same study were published, the article with the most updated data was selected for use in this study. In the case of duplicate publications, only one publication was included.

**Table 1:** Design Characteristics of Prospective cohort Studies of Glycosylated Hemoglobin and Cardiovascular Outcomes and All-cause Mortality, 1974–2011**.** *

Study name	Country	Project	No. of subjects	Age(y)	Men(%)	Outcome	Follow-uptime, y	HbA1c variable		Adjusted covariates
								Continuous	Category	
Stratton et al.,2006 (47)	United Kingdom	UKPDS 75	4320	53	60	All-cause mortality,CVD, CHD, Stroke	10.4	**√**		Age, sex, BP, lipid, smoking,
Adler et al., 2002 (16)	United Kingdom	UKPDS 59	3834	60	60	PAD	6	**√**		Age, BP, lipid, smoking, BMI
Adler et al., 1999 (25)	United Kingdom	UKPDS 47	5063	53	59	CVD, CHD, Stroke	10–10.3		**√**	Age, sex
Stratton et al., 2000 (17)	United Kingdom	UKPDS 35	3642	60	53	Heart Failure	10.4	**√**		Age, sex, BP, lipid, smoking, albuminuria
Mattock et al., 1998 (48)	United Kingdom	London DiabetesClinic	146	59	56	All-cause mortalityCHD mortality	7	**√**		Age, sex, BP, lipid, albuminuria, duration of DM
Currie et al., 2010 (18)	United Kingdom	Primary care GPRD	47970	64	55	All-cause mortality	3.9–4.4		**√**	Age, sex, lipid, smoking, DM medication
Donnan et al. 2006 (58)	United Kingdom	Primary care	4569	54.7	52.6	CHD	9.5	**√**		Age, sex, BP, lipid, smoking, BMI, duration of DM
Moss et al., 1999 (49)	United States	Primary care (WESDR)	1370	64.4	43.6	PAD	14	**√**	**√**	Age, sex, BP
Hirai et al, 2008 (50)	United States	Primary care (WESDR)	1007	68.6	44.9	All-cause mortality,CVD, CHD, Stroke	16	**√**		Age, sex, BP, smoking, BMI, duration of DM
Selvin et al., 2005 (20)	United States	The ARIC study	1626	45–64	–	CHD	8–10	**√**	**√**	Age, sex, BP, lipid, smoking, BMI
Selvin et al., 2005 (23)	United States	The ARIC study	1635	45–64	–	Stroke	9		**√**	Age, sex, BP, lipid, smoking, BMI
Iribarren et al.,2001 (21)	United States	The Kaiser PermanenteMedical care	48 858	58	52	Heart Failure	2.2	**√**	**√**	Age, sex, BP, lipid, smoking, BMI, DM medication, duration of DM
Agewall et al.,1997 (51)	Sweden	Hypertensionintervention trial	94	67	100	CVD	6.3	**√**		Age, sex, BP, lipid, smoking, duration of DM
Gall et al., 1995 (52)	Denmark	Hvidore Hospital	328	54	61.6	All-cause mortality CVD	5.3	**√**		Age, BP
Kuusisto et al.,1994 (32)	Finland	Population registry,East Finland	229	69	32.3	CHD	3.5	**√**		Sex, BP, lipid, smoking, BMI, duration of DM
Lehto et al., 1996 (28)	Finland	Population registry,East and west Finland	1044	58	55	PAD	7	**√**		Age, sex
Lehto et al., 1996 (29)	Finland	Population registry,East and west Finland	1059	58	55	Stroke	7	√		Age, sex
Lehto et al., 1997 (30)	Finland	Population registry,East and west Finland	1059	58	55	CHD	7	**√**		Age, sex, lipid
VAN Hateren et al.,2011 (53)	Netherlands	ZODIAC-20(Primary care)	374	80	34.8	All-cause mortalityCVD mortality	10	**√**		Age, sex, BP, lipid, smoking, BMI, albuminuria, duration of DM
Landman et al., 2010 (19)	Netherlands	ZODIAC-11(Primary care)	1145	68.7	46	All-cause mortalityCVD mortality	5.8	**√**	**√**	Age, sex, BP, lipid, smoking, BMI, albuminuria, DM medication, duration of DM
Roselli della Rovere et al.,2003 (54)	Italy	Diabetic outpatientclinic	120	67	44	CVD	9	**√**		Age, sex, BP, lipid, smoking
Elley et al., 2008 (24)	New Zealand	Primary care	48 444	60	49	CVD	2.4	**√**	**√**	Age, sex, BP, lipid, smoking, BMI, albuminuria, duration of DM, ethnicity
Florkowski et al., 2001(27)	New Zealand	Christ church Hospital Diabetes Center	447	62	46.5	All-cause mortality CHD	6	**√**		Age, sex, BP, lipid, smoking, BMI, albuminuria, DM medication, duration of DM
Standl et al., 1996 (55)	Germany	Munich General practitioner Project	290	65	36	CVD	10	**√**		Age, BP, lipid, BMI
Yang et al., 2007 (56)	China	The prince of Wales Hospital	7209	57	45.5	Stroke	5.37	**√**		Age, sex, BP, lipid, smoking, albuminuria
Yang et al., 2008 (22)	China	The prince of Wales Hospital	6969	57	44.8	Stroke	5.36		**√**	Age, sex, BP, lipid, BMI, albuminuria
Yang et al., 2008 (57)	China	The prince of Wales Hospital	7067	57	45.4	HF	5.52	**√**		Age, sex, BP, lipid, smoking, albuminuria, duration of DM

* CHD, coronary heart disease; CVD, cardiovascular disease; HF, heart failure; PAD, peripheral arterial disease; DM, diabetes mellitus; BMI, body mass index; BP, blood pressure.

### Data Extraction and Quality Assessment

Data were extracted by two independent reviewers (ZY and GH) using standardized data abstraction forms. Disagreements between reviewers were resolved by repeated examination of the original articles and discussion until consensus was achieved. Information on surname of the first author, year of publication, country of origin, mean age, percentage of male of study participants, sample size, number of study participants included in the final analysis, duration of follow-up, outcomes, estimate of the risk of association, variables adjusted in the analyses was extracted. For assessment of study quality, we evaluate 6 major items of each study: 1) is the instrument for measuring GHb validated? 2) does GHb allow quantification as both continuous and categorized variables? 3) are the outcomes determined by the specified criteria (i.e., medical record) or physician’s or patient’s judgments such as registry, death certificate, questionnaire, and patients’ self-report? 4) is the total follow-up duration ≥5 years? 5) are major CVD risk factors for in the statistical analyses, such as age, sex, blood pressure (hypertension), dyslipidemia (or LDL/total cholesterol level), smoking, duration of diabetes, treatment, albuminuria, etc? and 6) are subjects lost-to-follow up excluded from the analysis?

**Figure 2:**
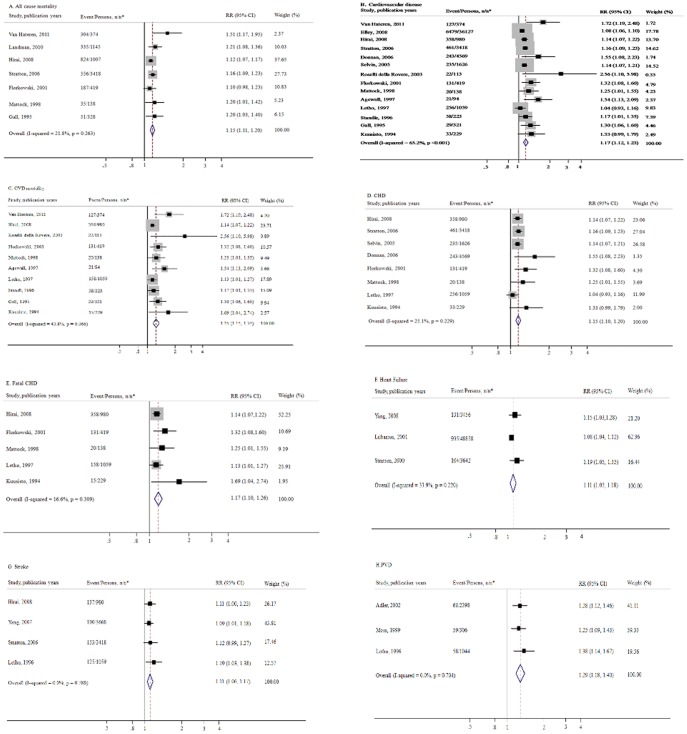
Forest plot of relative risks (RRs) and 95% confidence intervals (CIs) for the association between glycosylated hemoglobin and the main study outcomes risks in type 2 diabetes. CVD: cadiovascular diseases; CHD: conoary heart disease; PAD: peripheral arterial disease.

During data extraction, we abstracted adjusted relative risk (RR) for the association between GHb level either as a continuous or a categorical variable and the major outcomes (see below). Standard errors (SEs) for the estimates were abstracted or derived by using data reported in the original studies. When necessary, the original authors were contacted for additional information (3 authors contacted and 2 responded).

Reviewers recorded the following as the major outcomes of interest: all-cause mortality, incident CVD (non-fatal myocardial infarction, non-fatal stoke, and fatal CVD), CVD mortality, incident CHD (non-fatal myocardial infarction, and fatal CHD), CHD mortality, heart failure (non-fatal and fatal heart failure), incident stroke (non-fatal and fatal stoke), and PAD (lower-extremity peripheral arterial disease, amputation, and claudication).

### Data Synthesis and Analysis

Separate meta-analyses of the prospective cohort studies were carried out for the above major outcomes. All RRs estimates included in the pooled analyses were from the most fully adjusted multivariable models.

**Table 2:** Hazard ratios for cardiovascular outcomes risks according to glycosylated hemoglobin by different categories (shown by the first author and year of publication).*

	Cardiovascular outcomes	Categories	Categories(medians)	Hazard ratios(95% CI)	P Valuefor trend	Covariate in Multivariable Model								
						Age	Sex	BP	Lipid	Smoking	BMI orWHR	Albuminuria	DMMedications	Durationof DM
Selvin et al.,	CHD	<6.2	4.95	1	<0.001	√	√	√	√	√	√			
2005 (20)		5.2 to <5.7	5.45	1.24 (0.77–1.98)										
		5.7 to <6.5	6.10	1.57 (0.98–2.52)										
		6.5 to <8.2	7.35	2.04 (1.30–3.19)										
		≥8.2	9.05	2.37 (1.50–3.72)										
Landman et al.,	CVD mortality	<6.5	6.25	0.94 (0.47–1.91)		√	√	√	√	√	√	√	√	√
2010 (19)		6.5–7.0	6.75	1										
		7.0–8.0	7.5	1.4 (0.84–2.31)										
		8.0–9.0	8.5	1.71 (0.99–2.96)										
		>9.0	9.5	3.13 (1.62–6.05)										
Elley et al.,	CVD	<6.0	5.5	1	<0.001	√	√	√	√	√	√	√		√
2008 (24)		6.0 to <7.0	6.5	1.08 (0.97–1.19)										
		7.0 to <8.0	7.5	1.13 (1.02–1.25)										
		8.0 to <9.0	8.5	1.26 (1.12–1.41)										
		9.0 to <10.0	9.5	1.31 (1.15–1.50)										
		≥10.0	10.5	1.53 (1.34–1.73)										
Iribarren et al.,	Heart Failure	<7.0	6.5	1		√	√	√	√	√	√		√	√
2001 (21)		7.0 to <8.0	7.5	1.15 (0.93–1.43)										
		8.0 to <9.0	8.5	1.10 (0.88–1.38)										
		9.0 to <10.0	9.5	1.39 (1.11–1.74)										
		≥10.0	10.5	1.56 (1.26–1.93)										
Yang et al.,	Stroke	<6.0	5.55	0.77 (0.34–1.75)	0.531	√	√	√	√		√	√		
2008 (22)		6.0–6.9	6.45	1	reference									
		7.0–12.4	9.7	1.19 (0.71–1.99)	0.516									
		≥12.5	15.2	1.27 (0.17–9.68)	0.821									
Selvin et al.,	Stroke		5	1	<0.001	√	√	√	√	√	√	√		
2005 (23)			6	1.17 (0.62–2.19)										
			9	2.33 (1.29–4.21)										
Adler et al.,	CHD		6.9	1		√		√	√	√	√			
1999 (25)			9.1	1.20 (1.00–1.50)										
			11.3	1.40 (1.10–1.70)										
Adler et al.,	Stroke		6.9	1		√		√	√	√	√			
1999 (25)			9.1	1.10 (0.80–1.60)										
			11.3	1.00 (0.70–1.40)										

* CHD, coronary heart disease; CVD, cardiovascular disease; DM, diabetes mellitus; BMI, body mass index; BP, blood pressure; OR, hazard ratio; CI, confidence interval.

Most of the studies included in the present analysis reported the RRs of per unit change of GHb level, therefore, we converted studies that used different units in their original analyses based on the method previously published [15]. For example, there were 3 studies [28]–[30] that compared the RR for participants in the 3rd tertile of GHb to participants in the 2 lowest tertiles. In order to make these results comparable to the rest of studies, we assumed that there was a normal distribution for GHb values and used the reported mean and standard deviation (SD) to estimate the 33rd and 83rd percentiles of GHb (corresponding to the midpoints of the 2 lowest and the highest tertiles, respectively). Then, we divided the log RRs by the difference of these 2 values to estimate the effect of a 1% change in HbA1 [31]. Similarly, for one study [32] that compared the reported RR of above and below the median value of GHb, we estimated the effect of a 1% change but calculated the 25th and 75th percentiles instead.

After the RR estimate from each cohort study was converted to reflect a 1% increase in GHb [31], the pooled RRs and 95% confidence intervals (CIs) were calculated using the random-effects model. Statistical heterogeneity was assessed using the DerSimonian and Laird’s Q statistic and I^2^ statistic. The Q test provides information about the presence or absence of between-study heterogeneity, whereas the I^2^ statistic quantifies the degree of heterogeneity and is interpretable as the percentage of the total association that may be due to heterogeneity between studies (I^2^>50% was considered a meaningful level of heterogeneity). We also conducted a sensitivity analysis in which each prospective cohort study was excluded in turn to evaluate the influence of that prospective cohort study on the overall estimate. Publication bias was examined using funnel plots and Begg’s test. A meta-regression analysis was conduced to explore the sources of statistical heterogeneity in the meta-analyses. Subgroup analyses were conducted by stratifying the analysis according to studies that in different areas. All analyses were conducted by using STATA 10.0 (Stata Corporation, College Station, TX).

## Results

Of 3123 articles that were identified from the literature search, 3068 were excluded after an abstract or full-text review (Figure 1). Of the 55 articles for further review, 43 articles were relevant to GHb, macrovascular outcomes and all-cause mortality. Of these 43 articles, we excluded two that were not conducted in patients with type 2 diabetes [33], [34], two with follow-up time less than 1 year [35], [36], seven that were duplicated reports [5], [26], [37]–[41], one that had type 2 diabetes patients with heart failure at baseline [42], one with hemodialysis [43], and one with disable older women [44]. In addition, we excluded two nest case-control studies because of we were unable to perform the pooled analysis with other studies [45], [46]. Finally, 27 articles [16]–[25], [27]–[30], [32], [47]–[58] that reported 26 independent prospective cohorts were included in the present meta-analysis. Of them, two articles reported the same outcome with the same cohort study but using different analyses: one used continuous variable [56] and the other used categorical variable [22].

Table 1 summarizes the characteristics of the studies included in the present analysis. The sample size ranged from 94 to 48,858 participants, 10 studies (38%) had more than 3,000 patients of type 2 diabetes, and 3 studies [18], [21], [24] (12%) had more than 45,000. The mean follow-up time ranged from 2.2 to 16 years. The studies included were geographically heterogeneous: 5 were conducted in the United States (US), 7 in United Kingdom (UK), 4 in Finland, 2 in The Netherlands, 1 in Sweden, 1 in Denmark, 1 in Italy, 1 in Germany, 2 in New Zealand, and 2 in China. Most studies had primary care or clinic-based patient populations. Both men and women were included in 25 of the 26 studies; the remaining study included only men [51].

Most studies modeled the effect of baseline GHb measurements on the risk for incident CVD outcomes; however, 2 studies [47], [50] used updated mean GHb levels and modeled GHb as a time-dependent variable in the multivariable models.

The quality assessments of the included studies were summarized in the Supplementary table 1. The overall quality of included studies was good according to our 6-item evaluation criteria. All the studies adjusted for major CVD risk factors in the statistical analyses, had validated instrument for measuring GHb, had outcomes determined by specified criteria, and excluded participants who were lose during the follow-up (table S1). All studies had follow-up time longer than 1 year and only 4 studies had follow-up less than 5 years [18], [21], [24], [32]. Most studies treated GHb as continuous variables, and 5 studies treated GHb both as continuous and categorized variables in the analyses [19], [20], [21], [24], [49].

Figure 2 presents the individual and pooled RRs for all-cause mortality and cardiovascular outcomes. The pooled RR associated with a 1% increase in GHb level among patients with type 2 diabetes was 1.15 (95% CI, 1.11 to 1.20) for all-cause mortality in 7 independent studies, 1.17 (95% CI, 1.12 to 1.23) for incident CVD in 14 independent studies, 1.25 (95% CI, 1.15 to 1.35) for CVD mortality in 10 independent studies, 1.15 (95% CI, 1.10 to 1.20) for incident CHD in 8 independent studies, 1.17 (95% CI, 1.10 to 1.26) for fatal CHD in 5 independent studies, 1.11 (95% CI, 1.05 to 1.18) for incident heart failure in 3 independent studies, 1.11 (95% CI, 1.06 to 1.17) for incident stroke in 4 independent studies, and 1.29 (95% CI, 1.18 to 1.40) for incident PAD in 3 independent studies, respectively. The funnel plots and Begg’s test (figure S1) suggested that potential publication bias might be present for the CVD (P = 0.01) and CVD mortality (P = 0.004), but not for all-cause mortality (P = 0.07), CHD (P = 0.11), fatal CHD (P = 0.09), heart failure (P = 0.3), stroke (P = 0.09), and PAD (P = 0.2). In sensitivity analyses, exclusion of any single prospective cohort study from the analysis did not alter the overall findings of a positive association between GHb level and cardiovascular outcomes, and association between GHb level and all-cause mortality.

We also analyzed the heterogeneity among the studies of cardiovascular outcomes and all-cause mortality in persons with type 2 diabetes. The I^2^ statistics (P values for the Q test) in the above analyses were 21.8% (0.26) for all-cause mortality, 65.2% (<0.001) for CVD, 43.8% (0.07) for CVD mortality, 25.1% (0.23) for CHD, 16.6% (0.31) for fatal CHD, 33.9% (0.22) for heart failure, 0.0% (0.79) for stroke, and 0.0% (0.70) for PAD, respectively, which indicated no statistically significant heterogeneity for these pooled results except incident CVD.

To further investigate the potential sources of heterogeneity, we conducted subgroup analyses that compared the relative risk estimates for studies that adjusted for age, sex, blood pressure, and lipids with those that did not: for CVD, the pooled RR was 1.19 (95% CI, 1.11 to 1.27) for 9 studies adjusted for BP and lipids and 1.16 (95% CI, 1.05 to 1.29) for 5 studies that did not adjust for BP and lipids; for CHD, it was 1.17 (95% CI, 1.11 to 1.24; 5 studies adjusted for BP and lipids) vs.1.12 (95% CI, 1.03 to 1.21; 3 studies did not adjust for BP and lipids); for fatal CHD, it was 1.24 (95% CI, 1.06 to 1.46; 3 studies adjusted for BP and lipids) vs. 1.15 (95% CI, 1.08 to 1.21; 3 studies did not adjust for BP and lipids). In addition, we also conducted meta-regression and subgroup analyses to compare the RRs for studies that were conducted in different areas. For 6 outcomes (e.g. all-cause mortality, CVD, CVD mortality, CHD, fatal CHD, stroke) that allowed us to conduct meta-regression, area was not a significant factor that contributed to the heterogeneity (all P values >0.05).

Table 2 shows the individual RRs for cardiovascular outcomes according to categories of GHb levels. Eight studies reporting ≥3 categories of the GHb level were included. Among them, only 1 study reported both the case number and the total number of each category subgroup, thus we could not carry out the dose-response meta-analysis of the relation between GHb level and the cardiovascular outcomes due to the weight calculation. Except that 1 study showed no association [22], the results from the remaining 7 studies all suggested positive associations between GHb level and the cardiovascular outcomes with 3 studies reported significant P values (P<0.05) for testing the linear trend [23], [24].

Only two studies have evaluated the association between the categories of GHb level and the all-cause mortality and the results suggested a non-lineal association. One study in United Kingdom including 47,970 participants identified a “U-shape” association [18], while another study in the Netherlands including 1,145 participants detected an “almost positive” association [19]. With the limited numbers of the studies, we could not conduct the dose-response meta-analysis.

## Discussion

The meta-analysis of 26 prospective studies provides evidence that chronic exposure to increased glycemic level was associated with increased risks of all-cause mortality and cardiovascular outcomes in type 2 diabetes. We found that every 1% increase in GHb was associated with a 15% increase in hazard of all-cause mortality, 25% in CVD mortality, 17% in CVD, 15% in CHD, 17% in fatal CHD, 11% in heart failure, 11% in stroke, and 29% in PVD event. From the data of 8 prospective studies with 3 or more categories of GHb, we found a positive dose-response association, which provides additional support for our results of GHb level as a continuous variable and the major outcomes.

Our finding that an increased GHb level is associated with an increased risk of cardiovascular outcome is consistent with previous studies. This effect has been shown to be independent of other vascular risk factors. In a meta-analysis [15], Selvin et al. evaluated 10 prospective studies and concluded that every 1% increase in GHb was associated with a 18% increase in hazard of CVD, 13% in CHD, 16% in fatal CHD, and 17% in stroke incidence after controlling for potential confounders. These effect sizes are similar to those estimated in our study for CVD and CHD but higher for stroke. It may be due to the fewer stroke events (396) in the previous study. Our estimates from observational studies are highly consistent with the results from the RCTs. A meta-analysis of 5 RCTs showed, during 5-year treatment, reduction of HbA1c by 0.9% resulted in a 17% significant reduction in non-fatal MI events, 15% in CHD events, but non-significant reduction in stroke events in type 2 diabetic patients [12]. Another two meta-analyses of 4 or 5 relevant RCTs reported similar results, and also found non-significant reduction in HF events in type 2 diabetics [11], [13]. Ray et al. explained that the cases of stroke event in these RCTs were less than that myocardial infarction were reported, which may not have enough power to ascertain whether a significant benefit exists [12]. Due to the lack of case numbers and the total study sample sizes of each category of GHb, we could not conduct a dose-response analysis to identify the relationship between GHb level and the CVD outcomes; however, a positive association between GHb and CVD incident was suggested by carefully summarized findings from 8 studies (Table 2).

The relationship between GHb level and death has been studied, but the results are inconsistent. A previous meta-analysis of 10 prospective studies [15] did not have available data on all-cause mortality. Another meta-analysis of RCTs showed no association between GHb and CVD and all-cause mortality [11]–[13]. The short follow-up time, the small number of events, and the glucose-lowering drugs used in these clinical trials may actually have adverse cardiovascular effects, which would attenuate macrovascular benefits of improved glycemic control [59]. In the present study, a monotonic positive association between GHb level and the risks of all-cause mortality and CVD mortality was found. Every 1% increase in GHb was associated with a 16% increase in hazard of all-cause mortality, however we cannot assess the dose-response association between GHb and all-cause mortality due to the small number of prospective studies with 3 or more categories of GHb [18], [19]. Currie et al [18] found a U-shape association between the HbA1c level and all-cause mortality: low (6.4%) and high (10.6%) mean HbA1c values were associated with increased all-cause mortality as compared to median mean HbA1c values (7.5%) and this association was independent of the treatment regimen. Landman et al [19] detected an almost positive association between the HbA1c level and the risk of all-cause mortality. The results from RCTs [11]–[13] demonstrated that a low HbA1c therapeutic target level did not result in decreased mortality. The Action to Control Cardiovascular Risk in Diabetes (ACCORD) trial [60] was halted in February 2008 because of an unexpected excess of all-cause and CVD-related mortality in the intensive treatment group, suggesting that lowered HbA1c concentration might cause an excess risk for all-cause mortality. A clear adverse consequence of tight glycemic control was a 2 to 3 fold increased risk for severe hypoglycemia. These studies samples were composed of mostly elderly subjects with mean age at least 64 years. Nevertheless, these studies have important implications for clinical practice [60].

There are several biologically plausible mechanisms that might account for the finding that chronic hyperglycemia is associated with cardiovascular outcomes and all-cause mortality. Hyperglycemic periods play a major role in the activation of oxidative stress and overproduction of mitochondrial superoxide, which trigger various metabolic pathways of glucose-mediated vascular damage [61], [62]. Glucose can react with various proteins to form advanced glycation end products, which may contribute to long-term complications in diabetes, plaque formation, and atherosclerosis [59]. These effects are gradual and likely to be cumulative, occurring during decades of exposure to chronically elevated blood glucose levels. As explained by Selvin et al [15], this possibility suggests that most previous studies, including clinical trials, may have had insufficient follow-up to detect a moderate increase in risk.

This meta-analysis has notable strengths. We included many large studies with a correspondingly high number of incident cases, which improved the statistical power to detect significant differences. Our study was based on a comprehensive literature search. We believed that the inclusion of large studies, such as the 10-year post-trial monitoring of the UKPDS [47], and larger cohort studies conducted by Currie et al [18] in GPRD, Iribarren et al [21] in Kaiser Permanente Medical care, and Elley et al [24] in New Zealand, could potentially make our analysis more reliable. In addition, our sensitivity analysis showed minimal influence on the combined results for any single study, and the heterogeneity of variance between studies was allowed for by using random effects models. There are several limitations in this review. First, all studies were observational in nature and residual confounding cannot be totally ruled out. Second, the analysis was based on a single measurement of GHb, although GHb is indicative of time-averaged blood glucose concentration over the past 3 months [63]. Third, because of the lacking data, the dose-response relationships between GHb and CVD events and mortality cannot be estimated in the current analysis. Finally, as with any other systematic literature review, a limitation is a potential of publication bias, but the estimates in our study are similar to previous studies.

In conclusion, our results suggest that chronic hyperglycemia is associated with increased risks for cardiovascular outcomes and all-cause mortality among patients with type 2 diabetes and independent from other conventional risk factors. Our finding supports the notion that diabetic patients with higher GHb level should be closely followed due to their higher risks of cardiovascular outcomes and all-cause mortality. In addition, our data suggest that it might be desirable to achieve GHb as close to the normal glycemic range as possible.

## Supporting Information

Figure S1 **Funnel plots with 95% confidence limits for publication bias.** CVD: cadiovascular diseases; CHD: conoary heart disease; PAD: peripheral arterial disease; RR: relative risk; SE: standard error.
(TIF)

Table S1 Quality assessments* on prospective cohort studies on Glycosylated Hemoglobin Level in relation to Cardiovascular Outcomes and Death in Patients with Type 2 Diabetes.
(DOC)
