# Supplementary figures and images for: Glycosylated Hemoglobin in Relationship to Cardiovascular Outcomes and Death in Patients with Type 2 Diabetes: A Systematic Review and Meta-Analysis
Source: PLoS One. 2012 Aug 9;7(8):e42551. doi: 10.1371/journal.pone.0042551 (PMC3415427; doi:10.1371/journal.pone.0042551)

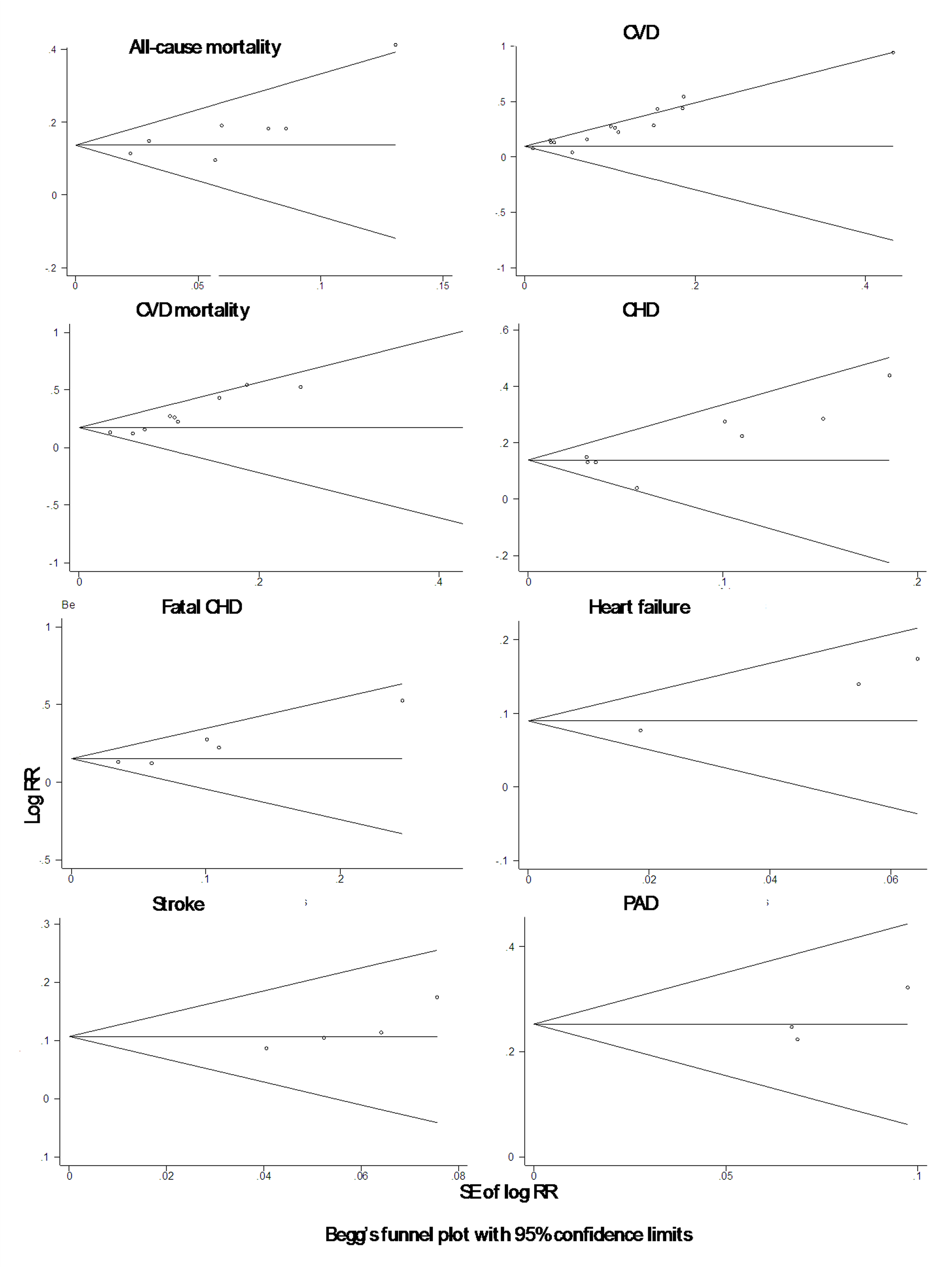

Supplement: Figure S1 — Funnel plots with 95% confidence limits for publication bias. CVD: cadiovascular diseases; CHD: conoary heart disease; PAD: peripheral arterial disease; RR: relative risk; SE: standard error. (TIF) [file pone.0042551.s001.tif]
