# Supplementary material for: Glycosylated Hemoglobin in Relationship to Cardiovascular Outcomes and Death in Patients with Type 2 Diabetes: A Systematic Review and Meta-Analysis
Source: PLoS One. 2012 Aug 9;7(8):e42551. doi: 10.1371/journal.pone.0042551 (PMC3415427; doi:10.1371/journal.pone.0042551)
Supplement: Table S1 — Quality assessments* on prospective cohort studies on Glycosylated Hemoglobin Level in relation to Cardiovascular Outcomes and Death in Patients with Type 2 Diabetes. (DOC) [file pone.0042551.s002.doc]

Table S1.

| Reference | 1. Is the instrument for measuring GHb validated? | 2. Does GHb allow quantification as both continuous and categorized variables? | 3. Are the outcomes determined by the specified criteria (i.e., medical record) or physician’s or patient’s judgments such as registry, death certificate, questionnaire, and patients’ self-report? | 4. Is the total follow-up duration ≥5 years? | 5. Are major CVD risk factors for in the statistical analyses?** | 6. Are subjects lost-to-follow up excluded from the analysis? | 7. Overall quality score |
| --- | --- | --- | --- | --- | --- | --- | --- |
| Stratton et al., 2006 (47) | 1 | 0 | 1 | 1 | 1 | 1 | 5 |
| Adler et al., 2002 (16) | 1 | 0 | 1 | 1 | 1 | 1 | 5 |
| Adler et al., 1999 (25) | 1 | 0 | 1 | 1 | 1 | 1 | 5 |
| Stratton et al., 2000 (17) | 1 | 0 | 1 | 1 | 1 | 1 | 5 |
| Mattock et al., 1998 (48) | 1 | 0 | 1 | 1 | 1 | 1 | 5 |
| Currie et al., 2010 (18) | 1 | 0 | 1 | 0 | 1 | 1 | 4 |
| Donnan et al. 2006 (58), | 1 | 0 | 1 | 1 | 1 | 1 | 5 |
| Moss et al., 1999 (49) | 1 | 1 | 1 | 1 | 1 | 1 | 6 |
| Hirai et al., 2008 (50) | 1 | 0 | 1 | 1 | 1 | 1 | 5 |
| Selvin et al., 2005 (20) | 1 | 1 | 1 | 1 | 1 | 1 | 6 |
| Selvin et al., 2005 (23) | 1 | 0 | 1 | 1 | 1 | 1 | 5 |
| Iribarren et al., 2001 (21) | 1 | 1 | 1 | 0 | 1 | 1 | 5 |
| Agewall et al., 1997 (51) | 1 | 0 | 1 | 1 | 1 | 1 | 5 |
| Gall et al., 1995 (52) | 1 | 0 | 1 | 1 | 1 | 1 | 5 |
| Kuusisto et al., 1994 (32) | 1 | 0 | 1 | 0 | 1 | 1 | 4 |
| Lehto et al., 1996 (28) | 1 | 0 | 1 | 1 | 1 | 1 | 5 |
| Lehto et al., 1996 (29) | 1 | 0 | 1 | 1 | 1 | 1 | 5 |
| Lehto et al., 1997 (30) | 1 | 0 | 1 | 1 | 1 | 1 | 5 |
| VAN Hateren et al., 2011 (53) | 1 | 0 | 1 | 1 | 1 | 1 | 5 |
| Landman et al., 2010 (19) | 1 | 1 | 1 | 1 | 1 | 1 | 6 |
| Roselli della Rovere et al., 2003 (54) | 1 | 0 | 1 | 1 | 1 | 1 | 5 |
| Elley et al., 2008 (24) | 1 | 1 | 1 | 0 | 1 | 1 | 5 |
| Florkowski et al., 2001(27) | 1 | 0 | 1 | 1 | 1 | 1 | 5 |
| Standl et al., 1996 (55) | 1 | 0 | 1 | 1 | 1 | 1 | 5 |
| Yang et al., 2007 (56) | 1 | 0 | 1 | 1 | 1 | 1 | 5 |
| Yang et al., 2008 (22) | 1 | 0 | 1 | 1 | 1 | 1 | 5 |
| Yang et al., 2008 (57) | 1 | 0 | 1 | 1 | 1 | 1 | 5 |
| % Studies scoring "Yes" | 100.0% | 18.5% | 100.0% | 85.2% | 100.0% | 100.0% | 5 |
